# Supplementary material for: Systematic analysis of the Hippo pathway organization and oncogenic alteration in evolution
Source: Sci Rep. 2020 Feb 21;10:3173. doi: 10.1038/s41598-020-60120-4 (PMC7035326; doi:10.1038/s41598-020-60120-4)
Supplement: Supplementary file 1 — Supplementary Information. [file 41598_2020_60120_MOESM1_ESM.pdf]

Systematic analysis of the Hippo pathway organization and oncogenic alteration in evolution

Yuxuan Chen, Han Han, Gayoung Seo, Rebecca Elizabeth Vargas, Bing Yang, Kimberly Chuc, Huabin Zhao and Wenqi Wang

Figure S1

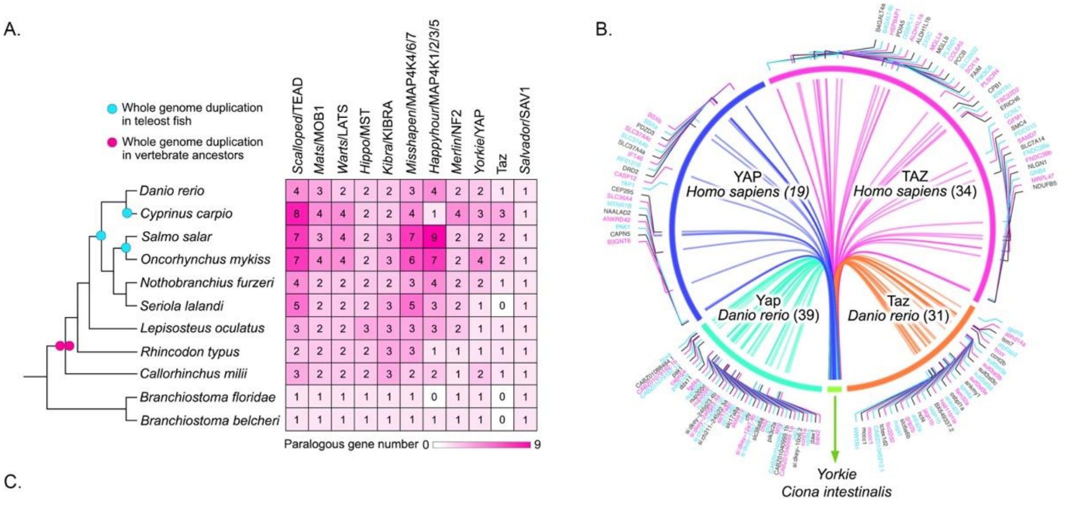

C.

Analysis of Type-I and Type-II functional divergences between YAP and TAZ

			Type I			Type II			
Gene	Cluster 1	Cluster 2	$\theta_I \pm$ Se[a]	LRT[b]	P value[c]	$\theta_{II} \pm$ Se[d]	z score[e]	LRT[b]	P value[c]
YAP/TAZ	YAP	TAZ	0.621352±0.138008	21.485551	3.56505E-06	0.039451±0.171655	0.22982727	0.039451±0.171655	0.409113

Notes:

[a] The maximum likelihood estimate of functional divergence coefficient (θ_I) with the standard error (SE).

[b] Maximum likelihood ratio estimated through type-I divergence analysis.

[c] The significance level estimated by chi-square test for type-I functional divergence and z-test for type-II functional divergence.

[d] The estimate of functional divergence coefficient (θ_{II}) with standard error.

[e] The ratio of θ_{II} to SE.

Figure S1. Analysis of the Hippo pathway paralogous gene history in fish. (This figure is related to Figure 1 and Table S1).

(**A**) The paralogous gene number of the Hippo pathway components is increased based on the rounds of whole genome duplication in fish. The Hippo pathway core components were searched in the genomes of *Branchiostomidae* and other indicated fish species by TBLASTN, where the human and mouse protein sequences were included as queries. The evolutionary points when the whole genome duplication occurred in teleost fish and vertebrate ancestors were marked as a blue dot and a red dot, respectively.

(**B**) The orthologous genes near *YAP* are matched with the ortholog genes near *TAZ* as compared between the genomes from *Homo sapiens* and *Danio rerio*. The syntenic analysis was performed by taking the regions near *Yorkie* in the *Ciona intestinalis* genome as an outgroup.

(**C**) The functional divergence test between YAP and TAZ. Both *p* value and parameters used in this test are listed as a table.

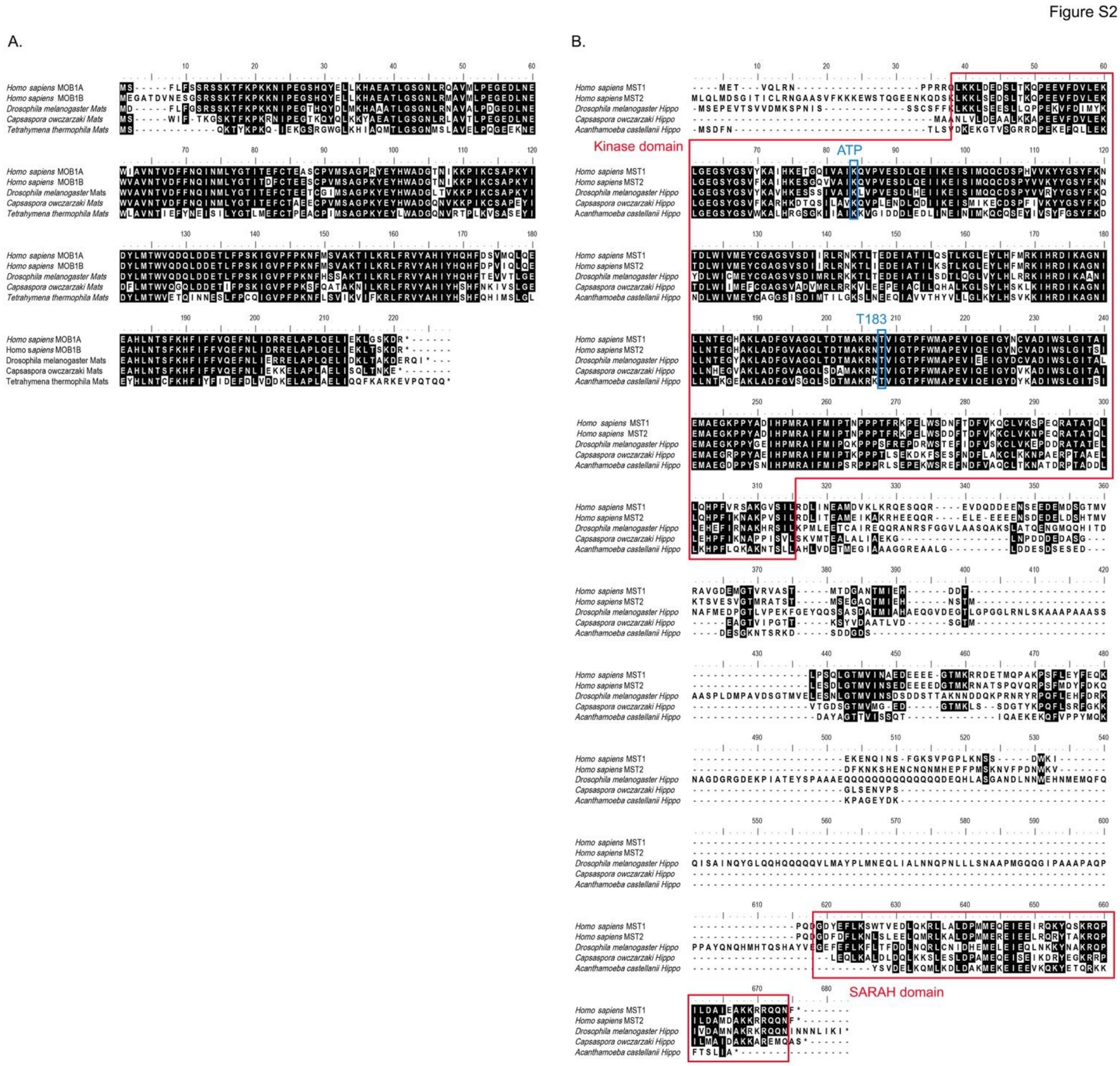

Figure S2. Sequence alignments for *Mats*/MOB1 and *Hippo*/MST proteins from the indicated species. (This figure is related to Figures 2 and 3).

(**A**) Sequence alignment for *Mats*/MOB1 is performed for its orthologous proteins in *Homo sapiens*, *Drosophila*, *Capsaspora owczarzaki* and *Tetrahymena thermophile*.

(**B**) Sequence alignment for *Hippo*/MST is performed for its orthologous proteins from *Homo sapiens*, *Drosophila*, *Capsaspora owczarzaki* and *Acanthamoeba castellanii*. The *Hippo*/MST kinase domain and SARAH domain were indicated in red. The *Hippo*/MST kinase ATP binding site and autophosphorylation site were indicated in blue.

Figure S3

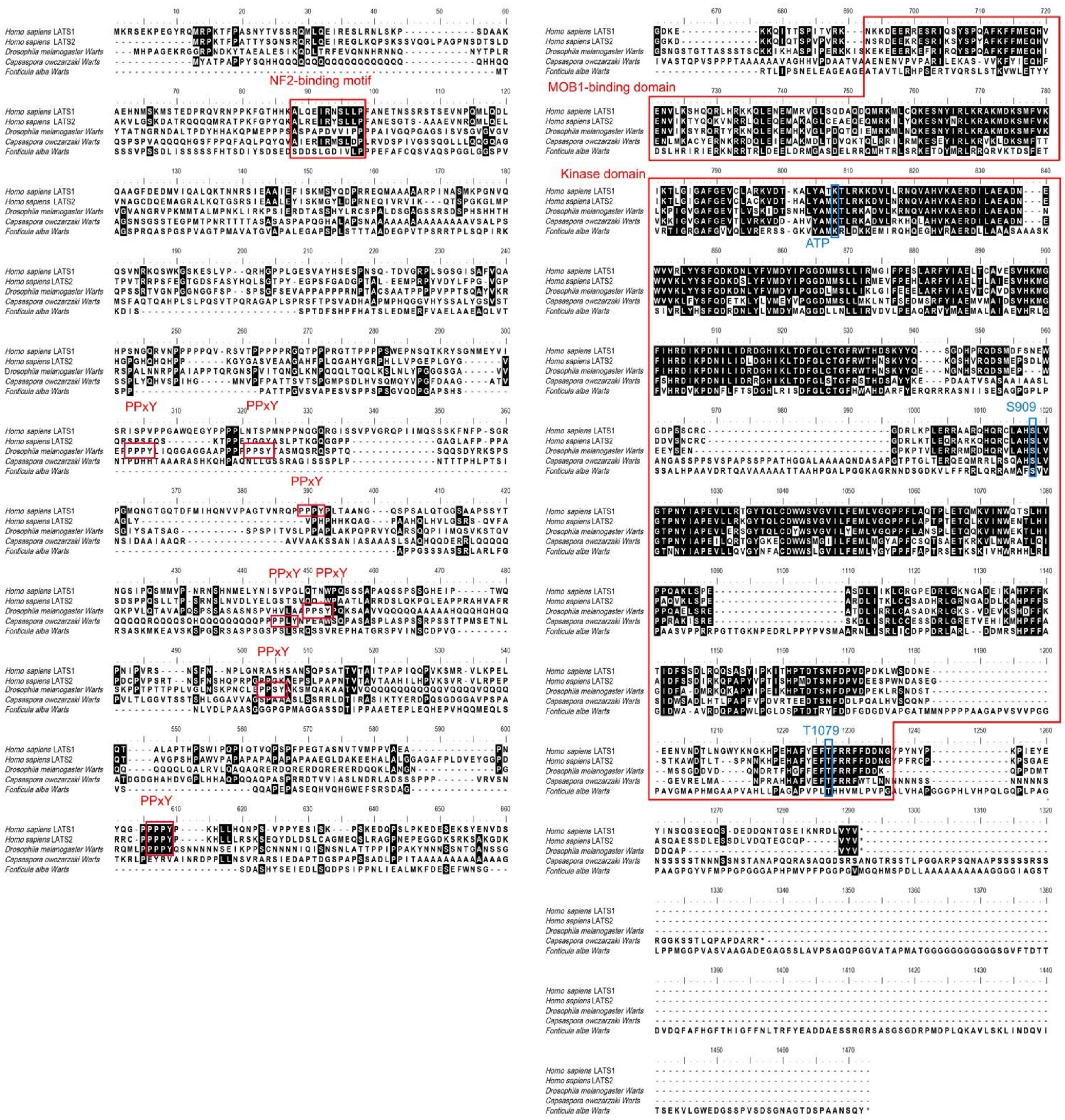

Figure S3. Sequence alignment for *Warts*/LATS proteins from the indicated species. (This figure is related to Figures 2 and 3).

Sequence alignment for *Warts*/LATS is performed for its orthologous proteins from *Homo sapiens*, *Drosophila*, *Capsaspora owczarzaki* and *Fonticula alba*. The *Warts*/LATS NF2-binding motif, PPxY motif, MOB1-binding domain and kinase domain were indicated in red. The *Warts*/LATS kinase ATP binding site, autophosphorylation site and phosphorylation site at its hydrophobic motif were indicated in blue.

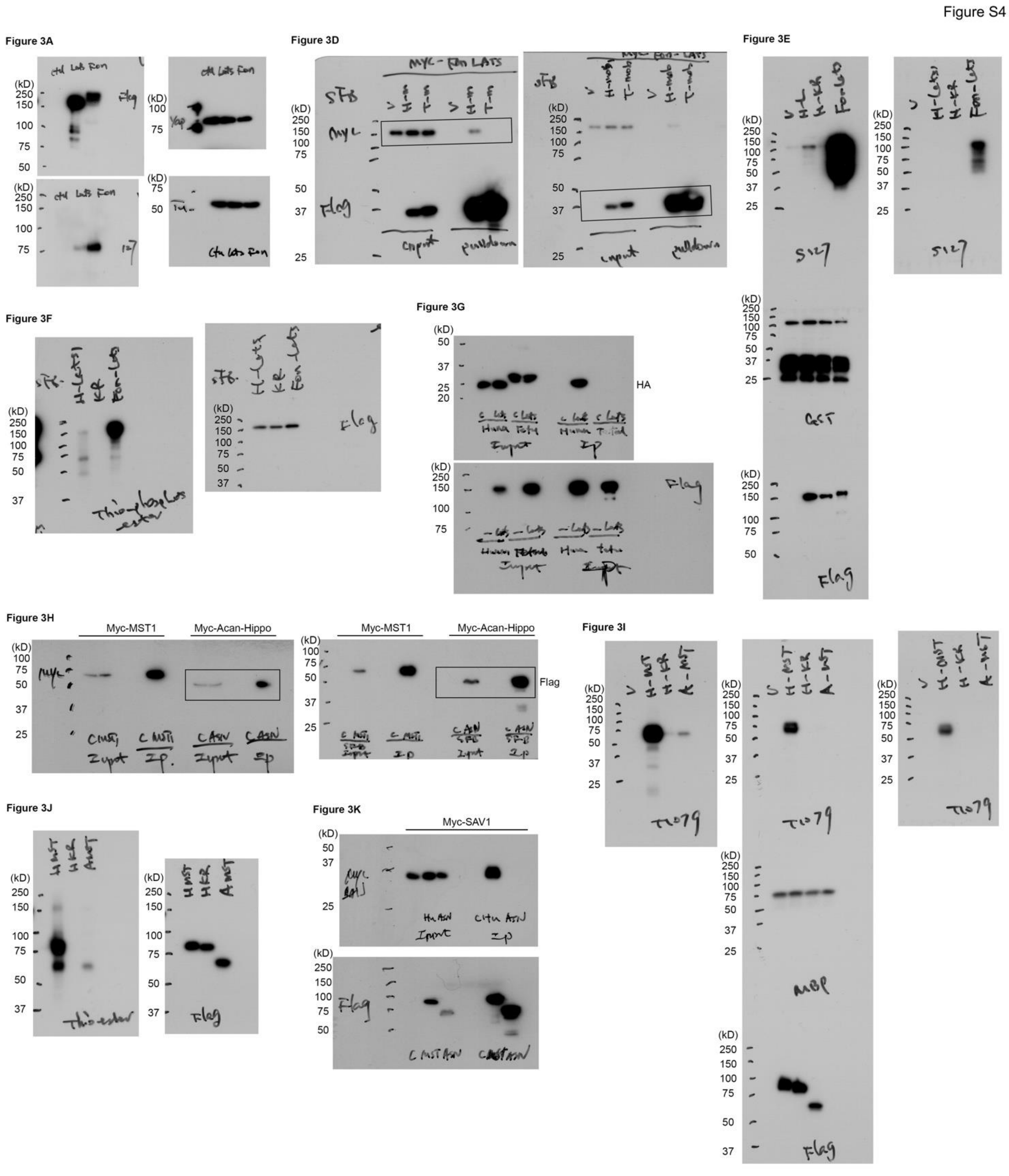

Figure S4. Uncropped Western blotting figures. (This figure is related to Figure 3).
